# Upfront Immunotherapy Approaches in the Management of Adults with Acute Lymphoblastic Leukemia

**DOI:** 10.3390/cancers17172746

**Published:** 2025-08-23

**Authors:** Moazzam Shahzad, Muhammad Kashif Amin, Talha Badar

**Affiliations:** 1 Division of Hematology and Oncology, Moffitt Cancer Center, Tampa, FL 33612, USA; moazzamshahzad1@gmail.com; 2 Division of Hematologic Malignancies & Cellular Therapeutics, The University of Kansas Medical Center, Kansas City, KS 66103, USA; 3 Division of Hematology and Medical Oncology, Mayo Clinic, Jacksonville, FL 32224, USA

**Keywords:** acute lymphoblastic leukemia, chemotherapy-free, immunotherapy, blinatumomab, inotuzumab ozogamicin, tyrosine kinase inhibitors, CAR-T cell therapy

## Abstract

The therapeutic landscape of adults with B-cell precursor acute lymphoblastic leukemia (BCP-ALL) is undergoing a paradigm shift, driven by the development of immunotherapy-based “chemo-free” and “chemo-light’ regimens. These strategies aim to achieve high efficacy with reduced toxicity, particularly in older adults who may not tolerate intensive chemotherapy. In Philadelphia chromosome-positive (Ph+) BCP-ALL, the incorporation of ABL tyrosine kinase inhibitors (TKIs) with blinatumomab (CD3/CD19 bispecific T-cell engager) has shown remarkable efficacy, with some studies reporting molecular response rates in the range of 90–100% and long-term survival exceeding 80% without the need for intensive chemotherapy or allogeneic hematopoietic cell transplantation (allo-HCT). In Philadelphia-negative (Ph−) BCP- ALL, an immunotherapy-based combination of blinatumomab and inotuzumab ozogamicin (anti-CD22 antibody-drug conjugate) has demonstrated high rates of complete remission and measurable residual disease (MRD) negativity, with manageable toxicity. While chimeric antigen receptor (CAR) T-cell therapy remains a transformative option for relapsed/refractory B-ALL, its integration into frontline treatment is still under investigation. Ongoing trials are evaluating the optimal sequencing and combinations of these agents and their potential to obviate the need for chemotherapy and/or allo-HCT in selected patients. As evidence continues to accumulate, chemo-free and chemo-light regimens, incorporating minimal chemotherapy with targeted agents to balance efficacy and reduced toxicity, are poised to redefine the standard of care for adults BCP-ALL, offering the possibility of durable remissions with reduced treatment-related morbidity.

## Simple Summary

Acute lymphoblastic leukemia (ALL) in adults is challenging to treat due to lower remission rates and higher toxicity from intensive chemotherapy, especially in older patients. This review explores recent advancements in immunotherapy-based strategies that aim to reduce or eliminate the need for traditional chemotherapy, thereby enhancing efficacy while minimizing side effects. By integrating targeted therapies like tyrosine kinase inhibitors and immunotherapies such as blinatumomab and inotuzumab ozogamicin, these approaches offer promising options for durable remissions and better quality of life. The findings could guide future treatments, potentially redefining standards for adult ALL management across age groups.

## 1. Introduction

Acute lymphoblastic leukemia (ALL) is a rapidly progressive hematologic malignancy characterized by the clonal proliferation of immature lymphoid progenitor cells, most commonly of B-cell origin and less frequently of T-cell origin [1]. It exhibits a characteristic bimodal age distribution, with the highest incidence observed in children aged 2–5 years and a second, smaller peak in adults over 50 [2]. While pediatric ALL is often highly curable, adult cases are associated with lower remission rates, higher relapse risk, and greater treatment-related toxicity, necessitating age-adapted therapeutic strategies [3,4]. The standard therapeutic approach for ALL includes induction, consolidation, and maintenance. In adults, standard regimens include hyper-CVAD (hyperfractionated cyclophosphamide, vincristine, adriamycin and dexamethasone), pediatric-inspired protocols (e.g., CALGB [Cancer and Leukemia Group B] 10403), and risk-adapted European strategies (e.g., UKALL 2011, GMALL [German Multicenter Acute Lymphoblastic Leukemia] 08/2013) [5,6]. For Philadelphia chromosome–positive (Ph+) ALL, *BCR::ABL1* directed tyrosine kinase inhibitors (TKIs) are added to improve durability of responses and survival [7,8]. Allogeneic hematopoietic cell transplantation (allo-HCT) remains central to curative therapy in high-risk or minimal residual disease (MRD)-positive patients [9]. However, adult patients, especially those ≥ 60 years, face significant challenges: increased toxicity, prolonged myelosuppression, and higher treatment-related mortality [10]. Response rates decline with age, and many adults cannot tolerate intensive regimens due to comorbidities, leading to interruptions or dose reductions. Additionally, prolonged hospitalizations can impact quality of life [11].

These limitations underscore the need for less toxic and more effective therapies. The development of chemo-free or chemo-sparing approaches has gained considerable momentum in recent years, driven by the need to improve tolerability while maintaining efficacy. These regimens integrate targeted agents such as TKIs, blinatumomab (a CD19-directed bispecific T-cell engager), and inotuzumab ozogamicin (InO; CD22-directed antibody-drug conjugate), either alone or in combination [12,13,14,15,16]. In Ph+ ALL, chemo-free regimens combining TKIs with blinatumomab have demonstrated high rates of complete molecular remission and durable disease control, even without the use of cytotoxic chemotherapy [16,17]. Chimeric antigen receptor (CAR)-T therapy, particularly anti-CD19 constructs, has shown promise in relapsed/refractory B-ALL and is being studied in earlier treatment lines [18,19,20,21].

As our understanding of leukemia biology and immunologic therapies deepens, chemo-free strategies hold the potential to shift the treatment paradigm in adult ALL, providing curative options with reduced toxicity and broader applicability across age groups. In this comprehensive review, we will explore the current landscape of chemo-free and chemo-sparing treatment strategies for ALL and highlight emerging approaches and future directions in this evolving field.

## 2. Methodology

**Figure 1:**
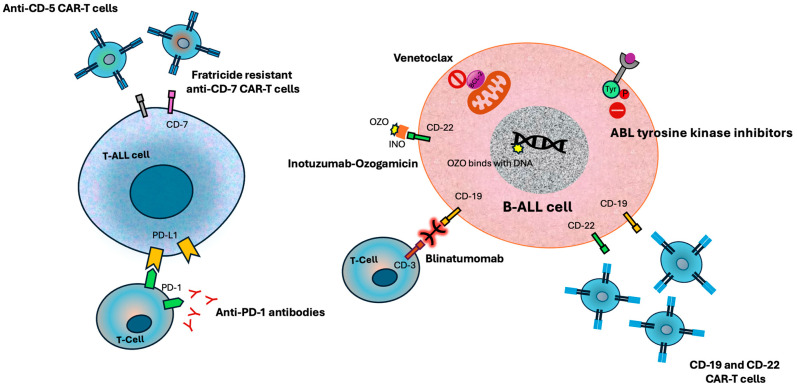
Graphical overview of immunotherapy and targeted approaches and their targets in acute lymphoblastic leukemia.

A comprehensive literature search was conducted across multiple databases to identify studies addressing chemo-free treatment strategies in ALL. The databases included PubMed, EMBASE, the Cochrane Library, ClinicalTrials.gov, and Google Scholar, covering literature published up to April 2025. Search terms consisted of combinations of “acute lymphoblastic leukemia,” “chemo-free,” “chemotherapy-free,” ‘chemo-light’, “targeted therapy,” “immunotherapy,” “blinatumomab,” “inotuzumab ozogamicin,” “CAR-T,” “tyrosine kinase inhibitors,” and “non-chemotherapy regimens,” utilizing Boolean operators to refine results. Eligible sources encompassed clinical trials, retrospective and prospective cohort studies, real-world data analyses, and review articles evaluating chemo-free or chemo-sparing regimens’ efficacy, safety, and feasibility in newly diagnosed or relapsed/refractory ALL. Studies involving pediatric, adolescent/young adult (AYA), and adult populations were included, with a particular focus on adult cohorts. Ongoing clinical trials registered on ClinicalTrials.gov were also reviewed to capture emerging therapies and future directions. Relevant studies were synthesized and discussed within the appropriate subsections of this review. In subsequent sections, we discuss various chemo-free options for managing ALL. Table 1 summarizes key clinical trials evaluating chemo-free and targeted immunotherapeutic strategies in ALL. Figure 1 provides a graphical overview of these therapeutic modalities and their cellular targets.

## 3. Ph− BCP ALL

### 3.1. Blinatumomab

Blinatumomab is a bispecific T-cell engager (BiTE) antibody construct that binds CD19 on B cells and CD3 on T cells, facilitating T-cell–mediated lysis of malignant B lymphoblasts [26].

It was approved for relapsed/refractory (R/R) Ph– BCP-ALL (TOWER) [27] and MRD-positive disease (BLAST) [28], enabling frontline use. In MRD-positive BCP-ALL, one cycle achieved 78% MRD negativity [28]. Recently, a phase 1b trial (NCT04521231) evaluated subcutaneous (SC) blinatumomab in 27 heavily pretreated adults with relapsed/refractory B-ALL [29]. At the 250 μg/500 μg dose, 85.7% achieved CR/CRh with 75% MRD-negativity; at 500 μg/1000 μg, 92.3% achieved CR/CRh with 100% MRD-negativity. Subcutaneous administration was well tolerated, and cytokine release syndrome (CRS) and neurologic events were manageable. Unlike the current intravenous blinatumomab, which requires a 28-day continuous infusion, necessitating patients to carry a portable pump, subcutaneous blinatumomab may offer a more convenient administration schedule, potentially reducing logistical burdens and improving quality of life while maintaining efficacy. However, these benefits require confirmation in ongoing trials.

Blinatumomab is often used as a bridge to allo-HCT in both MRD-positive and relapsed settings, with the goal of achieving remission and MRD clearance prior to transplant to improve long-term outcomes [30,31,32]. Another study evaluated whether blinatumomab is safe and effective as an alternative to intensive chemotherapy consolidation in children and young people with BCP-ALL who were chemotherapy-intolerant or chemotherapy-resistant. Patients who responded to blinatumomab received further chemotherapy and/or allo-HCT. This approach resulted in higher MRD negativity, fewer grade ≥3 AEs, and excellent 2-year disease-free survival (DFS;95.9%) and OS (97.8%), supporting a chemo-free consolidation strategy prior to allo-HCT in children and young adults (1–24 years) [33]. Blinatumomab’s superior efficacy and acceptable toxicity profile in R/R BCP-ALL, compared with chemotherapy, encouraged the use of blinatumomab upfront in combination with chemotherapy, with the intention of improving durability of responses and limiting chemotherapy exposure without compromising outcome. In a phase 2 study from the GIMEMA (Gruppo Italiano Malattie EMatologiche dell’Adulto) group, LAL2317, sequential chemotherapy, and blinatumomab were used to improve MRD negativity in adult Ph^–^ BCP-ALL [34]. Two cycles of blinatumomab were added to a pediatric-inspired chemotherapy backbone. The study enrolled 149 patients with a median age of 41 years (range, 18–65); at the end of induction, 88% of patients achieved CR, and 70% achieved MRD negativity, with MRD negativity increasing to 93% after the first cycle of blinatumomab. The disease-free survival (DFS) and OS at 3 years were 65% and 71%, respectively. The ECOG-ACRIN (Eastern Cooperative Oncology Group and American College of Radiology Imaging Network) led randomized phase 3 study (E1910; NCT02003222) showed blinatumomab + chemotherapy improved 3-yr OS to 85% vs. 68% with chemotherapy alone [35]. A more relevant study to chemo-reduced approaches is a phase 2 single-arm trial from MD Anderson Cancer Center, in which patients with newly diagnosed Ph+ B-ALL received up to 4 cycles of hyper-CVAD followed by 4 cycles of blinatumomab and maintenance therapy [36]. Among 38 patients (median age 37 years, range 18–59), 100% achieved CR/CRp/CRi, with 97% MRD negativity. The 3-year relapse-free survival rate was 70%, and the overall survival rate was 87%, comparing favorably with historical data for hyper-CVAD alone (3-year OS ~60%). This approach reduced chemotherapy exposure from the standard 8 cycles of hyper-CVAD to 4, demonstrating potential for chemo-sparing in frontline therapy.

In contrast to earlier studies, which administered blinatumomab during the consolidation phase, the Southwest Oncology Group led a single-arm Phase 2 trial evaluating blinatumomab induction/consolidation followed by POMP maintenance in older patients with BCP-ALL aged 65 years or older [12]. Blinatumomab induction was followed by 3 cycles of blinatumomab consolidation among responding patients and POMP maintenance for 18 months. Of 29 patients enrolled, 19 (66%) achieved CR, resulting in 3-year DFS and OS of 37%. Blinatumomab was well tolerated in adult patients, with no mortality observed during induction. Single-arm studies are showing promising outcomes when incorporating blinatumomab into frontline chemo-free induction strategies; however, it may not be an effective induction strategy among patients with a high-risk genomic profile or those with a high disease burden [37,38]. However, blinatumomab’s efficacy may be reduced in patients with high disease burden, as observed in real-world studies where higher tumor burden negatively impacted outcomes, which is a critical limitation since virtually every adult with newly diagnosed B-ALL presents with high disease burden by traditional measures. High-risk genomic profiles, such as TP53 mutations or complex karyotypes, may also limit blinatumomab’s effectiveness due to intrinsic resistance mechanisms and poorer T-cell function in these patients

### 3.2. Inotuzumab Ozogamicin (InO)

InO is a CD22-targeting antibody-drug conjugate used in various settings for BCP-ALL, particularly in adults [30,31]. Its most well-established use is in the relapsed/refractory (R/R) setting, as demonstrated by the pivotal phase III INO-VATE ALL trial [39]. This study compared InO with standard-of-care chemotherapy and showed significantly higher CR or CR with incomplete hematologic recovery (CR/CRi) rates (80.7% vs. 29.4%, *p* ≤ 0.001), longer median PFS (5.0 vs. 1.8 months, *p* ≤ 0.001), and a trend toward improved OS (7.7 vs. 6.2 months, *p* = 0.04).

Induction with intensive chemotherapy in older patients with BCP-ALL can be challenging, resulting in high treatment-related mortality, leading to dismal OS of 5 to 10 months [40]. The positive results with InO in the R/R setting prompted the development of an up-front combination of InO with low-intensity chemotherapy to improve outcome. A Phase 2 study from MD Anderson Cancer Center evaluated the combination of InO with a reduced-intensity chemotherapy regimen (mini-hyper-CVD) in newly diagnosed patients aged 60 years or older [41]. Among 74 evaluable patients, the response rate was 99% (CR in 89%), and MRD negativity was achieved in 94% overall. Protocol modifications, such as fractionated InO dosing and the addition of blinatumomab, were introduced mid-study to improve depth of response and reduce the risk of sinusoidal obstruction syndrome (SOS), which occurred in 8% of patients. With a median follow-up of 55 months, the 5-year overall survival was 47%, with outcomes particularly favorable in patients aged 60–69 years without poor-risk cytogenetics (5-year OS: 69%). Of note, 35 patients (44%) died in remission from secondary malignancies, infectious complications, SOS, and organ failure. This raises concerns about late toxicities potentially related, at least in part, to chemotherapy and InO combination.

The EWALL (European Working Group on Adult ALL) conducted a phase 2 study of InO with low-intensity chemotherapy in older patients (≥60 years) with newly diagnosed with Ph^–^ BCP-ALL [25]. The induction chemotherapy was delivered in 2 parts. Part 1 consisted of weekly vincristine and dexamethasone for 4 weeks, followed by 3 doses of InO (0.8 mg/m^2^ on day 1, 0.5 mg/m^2^ on days 8 and 15) and dexamethasone (20 mg on days 1–2, 8–9, 15–16, 22–23). Part 2 of induction, given to patients who achieved CR, comprised dexamethasone on day 1 and day 8, cyclophosphamide 300 mg/m^2^ from day 1 to day 3, and 2 doses of InO on day 1 and day 8 (0.5 mg/m^2^). Among 131 patients enrolled, the CR/CRi rate was 88.5% (57% MRD negative), and the 2-year OS was 54%. Although there is hope that moving immunotherapy to the front of the treatment course for patients with BCP-ALL will improve efficacy, the InO plus chemotherapy combination in older adults with BCP-ALL has shown that short- and long-term toxicity requires continued vigilance and reassessment of dosing and treatment sequencing.

The GMALL (German Multicenter Study Group for Adult ALL) group conducted a phase 2 trial (INITIAL-1) exploring up-front InO plus dexamethasone induction followed by age-adapted modified GMALL consolidation and maintenance chemotherapy in patients aged >55 years with BCP-ALL [13]. Among 43 patients with a median age of 64 years (range, 56–80) who received at least 2 cycles of InO, 100% achieved CR/CRi; 71% of which were MRD negative after the third cycle. After a median follow-up of 2.7 years, the estimated 3-year event -free survival (EFS) and OS were 55% (95% CI, 40–71) and 73% (95% CI, 59–87), respectively. Similarly, a Phase 2 study was conducted by the Alliance for Clinical Trials in Oncology (A041703) in older adults (≥60 years) with newly diagnosed Ph^–^CD22+ BCP-ALL who were ineligible for transplant, utilizing sequential InO induction followed by blinatumomab [14]. Blinatumomab was given for up to 3 cycles for patients refractory to InO or for 2 cycles if CR/CRi was achieved with InO. Among 31 eligible patients, the cumulative CR/CRi rates were 85% and 97% after InO alone and after InO plus blinatumomab, respectively. The one-year EFS and OS rates were 75% (95% CI, 61–92%) and 84% (95% CI, 72–98%), respectively.

InO is efficacious for BCP-ALL, with consistent activity across a range of disease burdens, unlike blinatumomab, which is less effective in high-burden settings [37,38,39,42]. A better treatment schema with hypofractionated doses of InO in combination with blinatumomab or chemotherapy may improve its safety without compromising efficacy in older BCP-ALL patients.

## 4. Ph+ BCP-ALL

Prior to *BCR::ABL1*-directed TKIs, Ph+ ALL had a dismal 5-year survival of ~20%, with allo-HSCT as the primary curative option despite high relapse rates [38]. Post-TKI, survival improves significantly (e.g., 3-yr OS 70–90% with ponatinib + blinatumomab), and chemo-free regimens may reduce the necessity of allo-HCT in low-risk patients, although it remains the standard for high-risk cases [8,9,24]. While ABL inhibitors with blinatumomab are standard, combinations with InO have been less explored, with limited data showing modest CR rates (e.g., 80% in small cohorts) due to higher toxicity risks like sinusoidal obstruction syndrome (SOS) [43]. Blinatumomab’s lower toxicity profile and synergy with TKIs make it preferred in Ph+ settings. Better outcomes in terms of depth of response and survival have been observed with third-generation TKIs, such as ponatinib, which offer broader activity against resistant mutations, including T315I, compared to first- and second-generation TKIs like imatinib and dasatinib [44].

Chemotherapy-free regimens combining TKI with immunotherapies are transforming the treatment landscape for Ph+ ALL. These approaches aim to reduce toxicity while maintaining high efficacy. First- and second-generation TKIs like imatinib and dasatinib target the BCR-ABL1 kinase domain, but third-generation TKIs like ponatinib offer broader activity against resistant mutations, including T315I, though with higher cardiovascular risk. Asciminib, an allosteric inhibitor that binds to the myristoyl pocket, offers a distinct mechanism with potentially improved tolerability and synergy in combination therapies; however, it is not yet widely approved for ALL. Several studies have evaluated dasatinib-based chemo-free strategies. The BLISSPHALL phase 2 trial combined corticosteroid induction with dasatinib and blinatumomab consolidation and maintenance in newly diagnosed Ph+ ALL patients. Of 17 patients, all achieved complete remission (CR), with 10 achieving complete molecular response (CMR). The regimen demonstrated high rates of MRD negativity, low relapse (2 patients), and minimal severe toxicity [45]. The GIMEMA D-ALBA phase 2 study employed dasatinib plus prednisone induction, followed by blinatumomab consolidation, in 63 patients with newly diagnosed Ph+ ALL. Ninety-eight percent achieved CR at the end of induction, with molecular response increasing from 29% to 93% after all blinatumomab cycles. At a median follow-up of 53 months, EFS and OS were 74.6% and 80.7%, respectively, though IKZF1 abnormalities and T315I mutations were associated with suboptimal outcomes [46]. The SWOG S1318 phase 2 trial evaluated dasatinib, prednisone, and blinatumomab in patients aged 65 and older with newly diagnosed Ph+ ALL. The regimen demonstrated a median OS of 6.5 years, offering a promising alternative for patients unsuitable for intensive chemotherapy [47].

Ponatinib-based approaches have also shown promise, particularly in addressing resistance to TKIs. In a phase 2 trial from MD Anderson, the combination of ponatinib + blinatumomab in 60 newly diagnosed Ph+ ALL patients achieved an 83% complete molecular response and 98% MRD negativity after a median follow-up of 24 months, with an estimated 3-year OS of 91%. The regimen reduced the need for chemotherapy and HSCT, though three patients experienced adverse events, leading to discontinuation of treatment [24]. The GIMEMA LAL2820 trial compared ponatinib + blinatumomab versus chemotherapy + imatinib in adults with newly diagnosed Ph+ ALL. Interim results showed superior MRD negativity (85% vs. 65%) and improved EFS (HR 0.45, *p* = 0.01) with ponatinib + blinatumomab, indicating that ponatinib may be preferred over imatinib for better depth of response and tolerability [48].

In another study, the combination of olverembatinib (available primarily in China) and blinatumomab in 13 Ph+ ALL patients resulted in a 100% 6-month OS and 87.5% EFS. Most patients proceeded to allo-HCT, with one relapse occurring after allo-HCT in a patient with the E255V mutation [49]. A phase 1 study of asciminib combined with dasatinib, prednisone, and blinatumomab in BCR-ABL1+ ALL reported promising preliminary results, with all 15 patients achieving CR and 93% MRD negativity after induction, and no dose-limiting toxicities, suggesting asciminib’s potential in chemo-free combinations due to its favorable safety profile [50]. These findings suggest that chemotherapy-free regimens combining TKIs with immunotherapies like blinatumomab can achieve high remission rates and may reduce the need for allo-HCT in Ph+ ALL patients. However, longer follow-up is necessary to confirm the durability of these responses.

While chemotherapy-free regimens are tremendously improving the outcome of Ph+ ALL, an increased incidence of central nervous system (CNS) relapse has been observed at long-term follow-up. In the D-ALBA study, 4 of 9 relapses were in the CNS, suggesting a need for a better CNS prophylaxis strategy [15]. An increasing course of intrathecal chemotherapy has been proposed to decrease the risk of CNS relapses with chemotherapy-free regimens, but its effectiveness needs further evaluation.

To validate the superior efficacy of upfront blinatumomab plus TKI combination compared with chemotherapy plus TKI, the ECOG-ACRIN cooperative group is conducting a randomized phase 3 study comparing outcomes of blinatumomab plus TKI (dasatinib or ponatinib) versus TKI plus hyper-CVAD chemotherapy in patients with treatment-naïve Ph+ ALL [51]. Similarly, the GIMEMA group is conducting a study comparing ponatinib plus blinatumomab induction with chemotherapy and imatinib, with the primary endpoint being EFS [52].

## 5. Chimeric Antigen Receptor-T Cell Therapy

CAR T-cell therapy targeting CD19 has become a cornerstone in the management of relapsed/refractory BCP-ALL [53,54]. Tisagenlecleucel, approved for patients ≤25 years, showed an 81% remission rate in the ELIANA trial, with all responders achieving MRD negativity. Long-term follow-up revealed a 5-year OS of 55% and durable remissions in 44% of early responders [54]. In the ZUMA-3 trial, brexucabtagene autoleucel (brexu-cel) demonstrated a 71% CR/CRi rate in adults with R/R B-ALL, with 97% MRD negativity among responders. The median remission duration was 12.8 months, relapse-free survival (RFS) 11.6 months, and median OS 18.2 months. Grade ≥3 CRS and neurotoxicity occurred in 24% and 25%, respectively, with two treatment-related deaths [55].

Obecabtagene autoleucel (obe-cel) in the FELIX trial reported a 77% CR/CRi rate with a favorable safety profile (grade ≥ 3 CRS 2.4%, neurotoxicity 7.1%) [56]. Recognizing that CAR T-cell therapy is more effective with lower disease burden, its integration into frontline treatment is being explored to achieve deep MRD negativity and reduce relapse without intensive chemotherapy. A phase 1/2 study of CD19/CD22 bispecific CAR T-cells in MRD-positive BCP-ALL, conducted in newly diagnosed patients after induction, reported a 100% response rate with 88% MRD-negative CR, suggesting potential for chemo-free consolidation in frontline settings [21]. This approach aims to shift CAR T-cell therapy earlier in the treatment paradigm, potentially reducing reliance on chemotherapy and allo-HCT while improving outcomes in newly diagnosed patients.

Considering CAR T-cell therapy appears to be more effective with lower disease burden, has recently been evaluated for the management of MRD-positive B-cell precursor acute lymphoblastic leukemia (BCP-ALL). In single-arm phase 1/2 studies, CD19 or CD19/CD22 bispecific CAR T-cells have demonstrated effectiveness in eradicating MRD without significant toxicities [20,21]. However, further evaluation is necessary to determine the cost-effectiveness of CAR T-cell therapy in improving survival.

## 6. Immunotherapy in T-Cell ALL

There are currently no approved chemotherapy-free options for T-cell ALL, particularly in the frontline setting, and outcomes with conventional chemotherapy in the relapsed/refractory (R/R) context remain poor. Investigational chemo-free strategies for T-ALL induction or consolidation are limited and not yet widely available, with most approaches still incorporating some chemotherapy, underscoring the challenge of developing fully chemo-free regimens for this disease. Emerging therapies are being explored to address this gap, though their clinical adoption remains distant.

One promising avenue involves CD7-targeted CAR-T cell therapy. A Phase 1 study of base-edited CD7 CAR-T cells in 12 patients with relapsed/refractory T-ALL or T-LBL showed a 100% complete remission rate by day 28 [57]. Over 6 months, 75% remained in remission. All patients experienced grade ≥3 cytopenias; 58% had infections, and 58% developed grade 1–2 CRS. No ICANS or GVHD occurred. Oh et al. treated 17 R/R T-ALL patients with autologous CD7 CAR-T cells modified using a protein expression blocker (PEBL) to prevent fratricide [58]. Sixteen achieved MRD-negative complete remission within one month. Toxicities were primarily mild, and durable remissions were observed in patients who subsequently underwent allogeneic stem-cell transplantation.

Another novel approach evaluated CD5-targeted CAR-T cells in 19 heavily pretreated R/R T-ALL patients, many of whom had relapsed after prior CD7 CAR-T therapy [59]. Sixteen received the infusion, and all achieved CR or CRi by day 30. However, long-term outcomes were mixed: among 12 patients who did not receive consolidation with transplant, several relapsed or died from infections. This highlights the therapy’s high initial response rate and the importance of consolidation strategies to mitigate late complications.

A case report highlighted the use of a chemo-free maintenance regimen combining a PD-1 inhibitor with a histone deacetylase inhibitor (HDACi) in an adult T-ALL patient [60]. HDAC inhibitors have shown anticancer activity in T-ALL by inducing apoptosis in leukemia cells. This combination may offer a targeted, less toxic strategy for selecting patients. However, chemo-free approaches for T-cell ALL induction or consolidation therapy, yet to be explored. This combination may offer a targeted, less toxic strategy for selecting patients. However, a prospective phase 2 study of pembrolizumab for MRD in ALL was terminated early due to insufficient activity, with only 1 of 12 patients converting to MRD-negative, highlighting the challenges in this approach [61]. The limited availability of these investigational therapies, combined with their reliance on subsequent chemotherapy or transplantation for sustained efficacy, underscores the urgent need for novel, fully chemo-free approaches that can be broadly implemented in T-ALL management.

## 7. Limitations and Challenges of Chemo-Free Regimens

While chemo-free and chemo-light regimens offer reduced toxicity compared to intensive chemotherapy, they present several side effects and limitations that warrant consideration, particularly in the absence of randomized controlled trials demonstrating superiority over traditional chemotherapy-based strategies [16]. Blinatumomab commonly causes cytokine release syndrome (CRS, 10–20% of cases with grade ≥3) and neurotoxicity (10–15% of cases with grade ≥ 3), often necessitating hospitalization for continuous infusion and dose interruptions, which complicates treatment delivery [27]. Inotuzumab ozogamicin (InO) is associated with hepatotoxicity, including veno-occlusive disease (VOD), which occurs in 5–15% of patients, particularly those with prior liver damage or allogeneic hematopoietic cell transplantation (allo-HCT), necessitating fractionated dosing and careful monitoring [41]. Tyrosine kinase inhibitors (TKIs) like ponatinib carry cardiovascular risks, with arterial occlusive events occurring in 5–10% of patients, while dasatinib may cause pleural effusions in 20–30% of patients, impacting tolerability, especially in older patients [44]. Asciminib, a newer TKI, shows a more favorable safety profile but requires further evaluation for long-term toxicities [50]. Chimeric antigen receptor (CAR) T-cell therapy poses risks of severe CRS (up to 40% grade ≥ 3) and neurotoxicity (up to 30% grade ≥ 3), with high costs and manufacturing delays limiting accessibility, particularly in resource-constrained settings [55]. Antigen loss (CD19 or CD22) leads to relapse in 20–40% of cases across these therapies, limiting salvage options for relapsed patients [16]. A higher incidence of central nervous system (CNS) relapse is observed, as seen in the D-ALBA study, where 4 of 9 relapses were CNS-involved, underscoring the need for enhanced CNS prophylaxis, such as increased intrathecal therapy, though its effectiveness remains unconfirmed [16]. The high cost of these therapies, significantly exceeding that of standard chemotherapy, poses economic challenges, particularly in low-resource settings, potentially impacting accessibility and healthcare economics [50]. The lack of long-term data and randomized trials comparing these regimens to chemotherapy-based approaches further limits their adoption as standard care. Optimizing patient selection, refining dosing schedules, developing strategies to enhance CNS penetration, and addressing antigen escape are critical to overcoming these challenges and improving long-term outcomes.

## 8. Additional Ongoing Trials and Future Directions

Future directions for blinatumomab in BCP-ALL focus on enhancing efficacy through more coherent drug combinations, expanding its use across disease stages and subtypes, and optimizing treatment sequencing. Combination regimens with InO and venetoclax (BCL-2 inhibitor) are actively explored to deepen responses and improve remission durability, particularly in relapsed/refractory and high-risk patients [23,42]. In Ph+ ALL, trials evaluate blinatumomab alongside with potent TKIs such as ponatinib, asciminib, and olverembatinib [42,49]. Several studies target MRD-positive patients in both frontline and post-transplant settings, leveraging blinatumomab’s T-cell-engaging mechanism to prevent overt relapse. Blinatumomab has also been tested as a bridge to both autologous and allogeneic stem-cell transplantation, with some trials incorporating innovative “sandwich” strategies where blinatumomab is administered between chemotherapy cycles to enhance MRD clearance. Additionally, pediatric studies investigate age-specific dosing and efficacy, while new approaches, such as short-course regimens and subcutaneous delivery, aim to reduce the treatment burden and improve patient convenience. Collectively, these trials reflect a shift toward more personalized, less toxic, and strategically timed immunotherapy use in ALL. A list of ongoing trials is provided in Table 2.

Trials like NCT03808610 (venetoclax + low-intensity mini-hyperCVD in newly diagnosed high-risk ALL) and NCT03319901 (venetoclax + mini-hyperCVD in newly diagnosed/R/R ALL) have reported results and aim to establish venetoclax in frontline settings [42,62]. In NCT03808610, among 20 patients (median age 62 years), the CR/CRi rate was 95%, with 85% MRD negativity and a 2-year OS of 80%. In NCT03319901, among 17 patients (median age 35 years), the CR/CRi rate was 82%, with 71% MRD negativity and a 1-year OS of 71%. Frontline venetoclax shows promise in T-ALL or in cases where immunotherapy has failed in B-ALL, due to its lack of reliance on surface antigen targeting. From our perspective, pediatric adoption awaits further trials due to higher chemo tolerability in younger patients.

Investigational CAR-T products are also advancing. CD22-targeted CAR T-cell therapies have been explored for patients relapsing after CD19-directed treatments. In a phase 1b trial of 16 heavily pretreated patients with R/R B-ALL, CD22 CAR T-cells achieved a 75% CR rate [63]. However, the median duration of response was limited to 77 days, and relapses were associated with CD22 downregulation, highlighting challenges with antigen modulation. To overcome antigen escape, dual-target CAR T-cell therapies targeting CD19 and CD22 have been developed. In a Phase 1 trial of 17 patients with R/R B-ALL, bispecific CD19/CD22 CAR T-cells achieved a 100% response rate, with 88% of patients achieving MRD-negative CR [6]. However, some relapses involved CD19-negative or low-expression disease, highlighting the need for improved CAR T-cell persistence. In a comparative study, tandem CD19/CD22 CAR T-cell therapy demonstrated superior efficacy over single-target CD19 CAR T-cells, with higher complete remission rates (95.9% vs. 82.7%; *p* = 0.012) and MRD-negative CR rates (81.6% vs. 66.7%; *p* = 0.092), along with a lower incidence of severe cytokine release syndrome (18.4% vs. 25.0%; *p* = 0.641) [64]. A meta-analysis of 10 trials (n = 194) reported a pooled CR rate of 87% for dual-targeted therapies, exceeding the 75% CR rate observed with CD22-directed CAR T-cell therapy alone, along with significantly higher MRD-negative response rates [65]. For T-ALL, the ongoing ECOG-ACRIN study with daratumumab for MRD-positive disease could provide a truly chemotherapy-free approach [66].

Beyond current trials, future advancements aim to address unmet needs through innovative delivery systems and biomarker-driven strategies. Novel formulations, such as nanoparticle-based delivery for bispecific antibodies or CAR T-cells, are being developed to enhance tissue penetration and reduce off-target effects, potentially improving efficacy in sanctuary sites like the central nervous system [67]. Biomarker-driven approaches, leveraging proteomic and transcriptomic profiling, are poised to identify novel therapeutic targets and predict resistance patterns, enabling tailored regimens for high-risk subgroups like those with Philadelphia-like ALL [68]. The global implementation of these therapies faces challenges, including regulatory harmonization and training healthcare systems for complex treatments such as CAR T-cell therapy. Strategies to overcome these barriers include establishing regional treatment hubs and leveraging telemedicine for patient monitoring, ensuring equitable access to advanced therapies [69]. These efforts, combined with ongoing trials, promise to further reduce reliance on chemotherapy, enhance patient outcomes, and address disparities in ALL care worldwide.

## 9. Conclusions

Chemo-free and chemo-light approaches are increasingly transforming the therapeutic landscape of adult BCP-ALL, particularly among older adults and patients with high-risk features unfit for intensive chemotherapy. The incorporation of targeted agents such as TKIs, blinatumomab, InO, and CAR T-cell therapy has demonstrated promising efficacy with reduced toxicity compared to conventional regimens. These strategies offer a more tailored and less toxic treatment alternative, potentially improving both survival and quality of life. Nonetheless, several critical questions remain, including optimal patient selection, sequencing or combination of therapies, and identification of predictive biomarkers for response and resistance. Ongoing and future clinical trials are expected to address these gaps and guide the integration of these novel therapies into standard clinical practice.

## Figures and Tables

**Table 1:** Key studies on upfront chemotherapy-free and chemotherapy-light approaches in the management of precursor B-cell acute lymphoblastic leukemia.

Immunotherapy and Combination	Study Phase	Ph Status	Sample Size	Median Age (Range), Years	CR Rates	MRD Negativity	Allo-HCT	Survival
Blinatumomab Monotherapy [22]	2	Ph+	45	55 (23–78)	CR/CRh: 36%	88% of responders	9 (20%)	OS: 9.0 mo; RFS: 6.8 mo (CR/CRh); MRD CR NR
Dasatinib + Blinatumomab (MRD-adapted) [16]	2	Ph+	63	54 (24–82)	98%	81% CMR after 2 cycles, 93% after 4 cycles	24 (38%)	3-yr OS: 84%
Inotuzumab + Blinatumomab (InO induction → Blina consolidation) [23]	2	Ph−	33	71 (60–84)	97% (CR: 58%, CRh: 36%, CRi: 3%)	NR	1 (3%)	1-yr OS: 84%; 1-yr EFS: 75%
Blinatumomab (steroids followed by POMP maintenance) [12]	2	Ph−	29	75 (66–84)	66% CR	92%	NR	3-yr DFS 37%, 3-yr OS 37%
Blinatumomab + Ponatinib [24]	2	Ph+	60	57 (38–72)	95% CR	98% CMR	6 (15%)	3-yr EFS 81%, OS 91%
Inotuzumab (dexamethasone induction followed by modified GMALL consolidation) [13]	2	Ph−	43	64 (56–80)	100% CR/CRi	71% after third cycle	NR	3-yr EFS 55%, OS 73%
Inotuzumab (low-intensity chemotherapy: vincristine, cyclophosphamide, dexamethasone) [25]	2	Ph−	131	68 (55–84)	88.5% CR/CRi	57%	NR	2-yr OS 54%

Abbreviations: CR: complete remission; CRh: CR with partial hematologic recovery; CRi: CR with incomplete hematologic recovery; MRD: measurable residual disease; OS: overall survival; RFS: relapse-free survival; EFS: event-free survival; Ph+: Philadelphia chromosome-positive; Ph−: Philadelphia chromosome-negative; POMP: 6-Mercaptopurine + vincristine + Methotrexate + prednisone; InO: inotuzumab ozogamicin; Blina: blinatumomab; allo-HCT: allogeneic hematopoietic cell transplantation; CMR: complete molecular remission; NR: not reported or not reached. Note: MRD negativity for dasatinib + blinatumomab [16] is after all blinatumomab cycles; MRD-adapted indicates treatment tailored based on measurable residual disease response.

**Table 2:** Ongoing trials for chemotherapy-free and chemo-light approaches in the management of B-cell precursor acute lymphoblastic leukemia.

NCT Number	Phase	Setting	Study Description	Sponsor	Status
NCT02143414	3	ND	Blinatumomab vs. Standard Chemotherapy in Ph+ ALL	Amgen	Active, not recruiting
NCT06649006	2	R/R	Intravenous Blinatumomab in Ph– B-ALL	Amgen	Recruiting
NCT06554626	1/2	ND	Blinatumomab + Venetoclax Sequenced with InO in B-ALL	Zhejiang University	Recruiting
NCT06507514	2	ND	Blinatumomab + Autologous HSCT Sandwich Strategy in B-ALL	Soochow University	Recruiting
NCT06308588	1	ND	Blinatumomab + Asciminib in Ph+ ALL	MD Anderson Cancer Center	Recruiting
NCT06287229	2	R/R	Brexucabtagene Autoleucel Consolidation Post Mini-HCVD/InO/Blinatumomab	MD Anderson Cancer Center	Recruiting
NCT06237192	2	ND	Blinatumomab (B-ALL) or Venetoclax (T-ALL) in Ph– ALL	National Research Center for Hematology, Russia	Recruiting
NCT06220487	1/2	ND	Olverembatinib + CD3/CD19 BiTE + Chidamide in Ph+ ALL	Nanfang Hospital	Recruiting
NCT06111625	2	ND	Short-term Blinatumomab as Bridge to Allo-HSCT in B-ALL	Sichuan University	Active, not recruiting
NCT06061094	2	ND	Chemotherapy + TKIs + Blinatumomab ± SCT in Ph+ ALL	Goethe University	Recruiting
NCT05559450	2	ND	Blinatumomab as Bridge to Allo-HSCT in High-Risk BCP-ALL	Soochow University	Recruiting
NCT04530565	2/3	ND	Steroids/TKIs + Blinatumomab vs. Chemotherapy in BCR-ABL+ ALL	NCI	Recruiting
NCT04722848	3	ND	Ponatinib + Blinatumomab vs. Chemotherapy + Imatinib in Ph+ ALL	GIMEMA	Recruiting

Abbreviations: ALL: acute lymphoblastic leukemia; B-ALL: B-cell ALL; BCP-ALL: B-cell precursor ALL; BiTE: bispecific T-cell engager; Brexu-cel: brexucabtagene autoleucel; Dex: Dexamethasone; HR: high-risk; InO: inotuzumab ozogamicin; NCT: National Clinical Trial; Ph+: Philadelphia chromosome-positive; ND: Newly diagnosed; Ph–: Philadelphia chromosome-negative; R/R: relapsed/refractory; SCT: stem-cell transplant; T-ALL: T-cell ALL; TKIs: tyrosine kinase inhibitors; Ven: venetoclax (BCL-2 inhibitor). (Listed in alphabetical order).

## Data Availability

No new data were created or analyzed in this study. Data sharing is not applicable to this article.

## References

[1] Terwilliger T., Abdul-Hay M. (2017). Acute lymphoblastic leukemia: A comprehensive review and 2017 update. Blood Cancer J..

[2] Dores G.M., Devesa S.S., Curtis R.E., Linet M.S., Morton L.M. (2012). Acute leukemia incidence and patient survival among children and adults in the United States, 2001–2007. Blood.

[3] Samra B., Jabbour E., Ravandi F., Kantarjian H., Short N.J. (2020). Evolving therapy of adult acute lymphoblastic leukemia: State-of-the-art treatment and future directions. J. Hematol. Oncol..

[4] Badar T., Luger S.M., Litzow M.R. (2025). Incorporation of immunotherapy into frontline treatment for adults with B-cell precursor acute lymphoblastic leukemia. Blood.

[5] Rowe J.M., Buck G., Burnett A.K., Chopra R., Wiernik P.H., Richards S.M., Lazarus H.M., Franklin I.M., Litzow M.R., Ciobanu N. (2005). Induction therapy for adults with acute lymphoblastic leukemia: Results of more than 1500 patients from the international ALL trial: MRC UKALL XII/ECOG E2993. Blood.

[6] Siegel S.E., Advani A., Seibel N., Muffly L., Stock W., Luger S., Shah B., DeAngelo D.J., Freyer D.R., Douer D. (2018). Treatment of young adults with Philadelphia-negative acute lymphoblastic leukemia and lymphoblastic lymphoma: Hyper-CVAD vs. pediatric-inspired regimens. Am. J. Hematol..

[7] Badar T., Alkhateeb H., Aljurf M., Kharfan-Dabaja M.A. (2023). Management of Philadelphia chromosome positive acute lymphoblastic leukemia in the current era. Curr. Res. Transl. Med..

[8] Hoelzer D., Bassan R., Boissel N., Roddie C., Ribera J.M., Jerkeman M. (2024). ESMO Clinical Practice Guideline interim update on the use of targeted therapy in acute lymphoblastic leukaemia. Ann. Oncol..

[9] Fenske T.S., Hamadani M., Cohen J.B., Costa L.J., Kahl B.S., Evens A.M., Hamlin P.A., Lazarus H.M., Petersdorf E., Bredeson C. (2016). Allogeneic Hematopoietic Cell Transplantation as Curative Therapy for Patients with Non-Hodgkin Lymphoma: Increasingly Successful Application to Older Patients. Biol. Blood Marrow Transpl..

[10] Luskin M.R. (2021). Acute lymphoblastic leukemia in older adults: Curtain call for conventional chemotherapy?. Hematol. Am. Soc. Hematol. Educ. Program..

[11] Gökbuget N. (2013). How I treat older patients with ALL. Blood.

[12] Advani A.S., Moseley A., O’Dwyer K.M., Wood B.L., Fang M., Wieduwilt M.J., Aldoss I., Park J.H., Klisovic R.B., Baer M.R. (2022). SWOG 1318: A Phase II Trial of Blinatumomab Followed by POMP Maintenance in Older Patients With Newly Diagnosed Philadelphia Chromosome–Negative B-Cell Acute Lymphoblastic Leukemia. J. Clin. Oncol..

[13] Stelljes M., Raffel S., Alakel N., Wäsch R., Kondakci M., Scholl S., Rank A., Hänel M., Spriewald B., Hanoun M. (2024). Inotuzumab Ozogamicin as Induction Therapy for Patients Older Than 55 Years With Philadelphia Chromosome–Negative B-Precursor ALL. J. Clin. Oncol..

[14] Wieduwilt M.J., Yin J., Kour O., Teske R., Stock W., Byrd K., Doucette K., Mangan J., Masters G.A., Mims A.S. (2023). Chemotherapy-free treatment with inotuzumab ozogamicin and blinatumomab for older adults with newly diagnosed, Ph-negative, CD22-positive, B-cell acute lymphoblastic leukemia: Alliance A041703. J. Clin. Oncol..

[15] Advani A.S., Moseley A., O’Dwyer K.M., Wood B.L., Park J., Wieduwilt M., Jeyakumar D., Yaghmour G., Atallah E.L., Gerds A.T. (2023). Dasatinib/prednisone induction followed by blinatumomab/dasatinib in Ph+ acute lymphoblastic leukemia. Blood Adv..

[16] Foà R., Bassan R., Elia L., Piciocchi A., Soddu S., Messina M., Ferrara F., Lunghi M., Mulè A., Bonifacio M. (2024). Long-Term Results of the Dasatinib-Blinatumomab Protocol for Adult Philadelphia-Positive ALL. J. Clin. Oncol..

[17] Jabbour E., Short N.J., Jain N., Huang X., Montalban-Bravo G., Banerjee P., Rezvani K., Jiang X., Kim K.H., Kanagal-Shamanna R. (2023). Ponatinib and blinatumomab for Philadelphia chromosome-positive acute lymphoblastic leukaemia: A US, single-centre, single-arm, phase 2 trial. Lancet Haematol..

[18] Othman T., Logan A.C., Muffly L., Leonard J., Park J., Shah B., Aldoss I. (2024). The Role of CAR T-Cell Therapy in Relapsed/Refractory Adult B-ALL. J. Natl. Compr. Cancer Netw..

[19] Badar T., Shah N.N. (2020). Chimeric Antigen Receptor T Cell Therapy for Acute Lymphoblastic Leukemia. Curr. Treat. Options Oncol..

[20] Lu W., Wei Y., Cao Y., Xiao X., Li Q., Lyu H., Jiang Y., Zhang H., Li X., Jiang Y. (2021). CD19 CAR-T cell treatment conferred sustained remission in B-ALL patients with minimal residual disease. Cancer Immunol. Immunother..

[21] Niu J., Qiu H., Xiang F., Zhu L., Yang J., Huang C., Zhou K., Tong Y., Cai Y., Dong B. (2023). CD19/CD22 bispecific CAR-T cells for MRD-positive adult B cell acute lymphoblastic leukemia: A phase I clinical study. Blood Cancer J..

[22] Martinelli G., Boissel N., Chevallier P., Ottmann O., Gökbuget N., Topp M.S., Fielding A.K., Rambaldi A., Ritchie E.K., Papayannidis C. (2017). Complete Hematologic and Molecular Response in Adult Patients With Relapsed/Refractory Philadelphia Chromosome-Positive B-Precursor Acute Lymphoblastic Leukemia Following Treatment With Blinatumomab: Results From a Phase II, Single-Arm, Multicenter Study. J. Clin. Oncol..

[23] Wieduwilt M., Yin J., Kour O., Teske R., Stock W., Byrd K., Doucette K., Mangan J., Masters G., Mims A. (2023). S117: Chemotherapy-free treatment with inotuzumab ozogamicin and blinatumomab for older adults with newly-diagnosed, PH-negative, CD22-positive, B-cell acute lymphoblastic leukemia: Alliance A041703. HemaSphere.

[24] Kantarjian H., Short N.J., Haddad F.G., Jain N., Huang X., Montalban-Bravo G., Kanagal-Shamanna R., Kadia T.M., Daver N., Chien K. (2024). Results of the Simultaneous Combination of Ponatinib and Blinatumomab in Philadelphia Chromosome-Positive ALL. J. Clin. Oncol..

[25] Chevallier P., Leguay T., Delord M., Salek C., Kim R., Huguet F., Hicheri Y., Wartiovaara-Kautto U., Raffoux E., Cluzeau T. (2024). Inotuzumab Ozogamicin and Low-Intensity Chemotherapy in Older Patients With Newly Diagnosed CD22(+) Philadelphia Chromosome-Negative B-Cell Precursor Acute Lymphoblastic Leukemia. J. Clin. Oncol..

[26] Nagorsen D., Bargou R., Ruttinger D., Kufer P., Baeuerle P.A., Zugmaier G. (2009). Immunotherapy of lymphoma and leukemia with T-cell engaging BiTE antibody blinatumomab. Leuk. Lymphoma.

[27] Kantarjian H., Stein A., Gökbuget N., Fielding A.K., Schuh A.C., Ribera J.-M., Wei A., Dombret H., Foà R., Bassan R. (2017). Blinatumomab versus Chemotherapy for Advanced Acute Lymphoblastic Leukemia. N. Engl. J. Med..

[28] Gökbuget N., Zugmaier G., Dombret H., Stein A., Bonifacio M., Graux C., Faul C., Brüggemann M., Taylor K., Mergen N. (2020). Curative outcomes following blinatumomab in adults with minimal residual disease B-cell precursor acute lymphoblastic leukemia. Leuk. Lymphoma.

[29] Jabbour E., Zugmaier G., Agrawal V., Martínez-Sánchez P., Rifón Roca J.J., Cassaday R.D., Böll B., Rijneveld A., Abdul-Hay M., Huguet F. (2024). Single agent subcutaneous blinatumomab for advanced acute lymphoblastic leukemia. Am. J. Hematol..

[30] Badar T., Szabo A., Dinner S., Liedtke M., Burkart M., Shallis R.M., Yurkiewicz I.R., Kuo E., Khan M.A., Balasubramanian S. (2021). Sequencing of novel agents in relapsed/refractory B-cell acute lymphoblastic leukemia: Blinatumomab and inotuzumab ozogamicin may have comparable efficacy as first or second novel agent therapy in relapsed/refractory acute lymphoblastic leukemia. Cancer.

[31] Badar T., Szabo A., Advani A., Wadleigh M., Arslan S., Khan M.A., Aldoss I., Siebenaller C., Schultz E., Hefazi M. (2020). Real-world outcomes of adult B-cell acute lymphocytic leukemia patients treated with blinatumomab. Blood Adv..

[32] Badar T., Szabo A., Litzow M., Burkart M., Yurkiewicz I., Dinner S., Hefazi M., Shallis R.M., Podoltsev N., Patel A.A. (2021). Multi-institutional study evaluating clinical outcome with allogeneic hematopoietic stem cell transplantation after blinatumomab in patients with B-cell acute lymphoblastic leukemia: Real-world data. Bone Marrow Transpl..

[33] Hodder A., Mishra A.K., Enshaei A., Baird S., Bhuller K., Elbeshlawi I., Bonney D., Clesham K., Cummins M., Vedi A. (2024). Blinatumomab for First-Line Treatment of Children and Young Persons With B-ALL. J. Clin. Oncol..

[34] Bassan R., Chiaretti S., Della Starza I., Santoro A., Spinelli O., Tosi M., Elia L., Cardinali D., De Propris M.S., Piccini M. (2025). Up-front blinatumomab improves MRD clearance and outcome in adult Ph- B-lineage ALL: The GIMEMA LAL2317 phase 2 study. Blood.

[35] Litzow M.R., Sun Z., Mattison R.J., Paietta E.M., Roberts K.G., Zhang Y., Racevskis J., Lazarus H.M., Rowe J.M., Arber D.A. (2024). Blinatumomab for MRD-Negative Acute Lymphoblastic Leukemia in Adults. N. Engl. J. Med..

[36] Jabbour E., Short N.J., Jain N., Thompson P.A., Kadia T.M., Ferrajoli A., Huang X., Yilmaz M., Alvarado Y., Patel K.P. (2022). Hyper-CVAD and sequential blinatumomab for newly diagnosed Philadelphia chromosome-negative B-cell acute lymphocytic leukaemia: A single-arm, single-centre, phase 2 trial. Lancet Haematol..

[37] Queudeville M., Stein A.S., Locatelli F., Ebinger M., Handgretinger R., Gökbuget N., Gore L., Zeng Y., Gokani P., Zugmaier G. (2023). Low leukemia burden improves blinatumomab efficacy in patients with relapsed/refractory B-cell acute lymphoblastic leukemia. Cancer.

[38] Cabannes-Hamy A., Brissot E., Leguay T., Huguet F., Chevallier P., Hunault M., Escoffre-Barbe M., Cluzeau T., Balsat M., Nguyen S. (2022). High tumor burden before blinatumomab has a negative impact on the outcome of adult patients with B-cell precursor acute lymphoblastic leukemia. A real-world study by the GRAALL. Haematologica.

[39] Kantarjian H.M., DeAngelo D.J., Stelljes M., Martinelli G., Liedtke M., Stock W., Gökbuget N., O’Brien S., Wang K., Wang T. (2016). Inotuzumab Ozogamicin versus Standard Therapy for Acute Lymphoblastic Leukemia. N. Engl. J. Med..

[40] Geyer M.B., Hsu M., Devlin S.M., Tallman M.S., Douer D., Park J.H. (2017). Overall survival among older US adults with ALL remains low despite modest improvement since 1980: SEER analysis. Blood.

[41] Kantarjian H., Ravandi F., Short N.J., Huang X., Jain N., Sasaki K., Daver N., Pemmaraju N., Khoury J.D., Jorgensen J. (2018). Inotuzumab ozogamicin in combination with low-intensity chemotherapy for older patients with Philadelphia chromosome-negative acute lymphoblastic leukaemia: A single-arm, phase 2 study. Lancet Oncol..

[42] Short N.J., Jabbour E., Jain N., Senapati J., Nasr L., Haddad F.G., Li Z., Hsiao Y.C., Yang J.J., Pemmaraju N. (2024). A phase 1/2 study of mini-hyper-CVD plus venetoclax in patients with relapsed/refractory acute lymphoblastic leukemia. Blood Adv..

[43] Jain N., Maiti A., Ravandi F., Konopleva M., Daver N., Kadia T., Pemmaraju N., Short N., Kebriaei P., Ning J. (2021). Inotuzumab ozogamicin with bosutinib for relapsed or refractory Philadelphia chromosome positive acute lymphoblastic leukemia or lymphoid blast phase of chronic myeloid leukemia. Am. J. Hematol..

[44] Jabbour E., Short N.J., Ravandi F., Huang X., Daver N., DiNardo C.D., Konopleva M., Pemmaraju N., Wierda W., Garcia-Manero G. (2018). Combination of hyper-CVAD with ponatinib as first-line therapy for patients with Philadelphia chromosome-positive acute lymphoblastic leukaemia: Long-term follow-up of a single-centre, phase 2 study. Lancet Haematol..

[45] Geyer M.B., Mascarenhas J., Smith M., Pascual S., Shah A., Silvestrone M.R., Czaplinska T., Johnson K., Thompson M.C., Park J.H. (2023). Chemotherapy-Sparing Induction Followed By Consolidation and Maintenance with Blinatumomab and Concurrent Oral Tyrosine Kinase Inhibitor Therapy for Newly-Diagnosed Philadelphia Chromosome-Positive Acute Lymphoblastic Leukemia: Primary Endpoint Results from the Blissphall Study. Blood.

[46] Foà R., Bassan R., Vitale A., Elia L., Piciocchi A., Puzzolo M.C., Canichella M., Viero P., Ferrara F., Lunghi M. (2020). Dasatinib-Blinatumomab for Ph-Positive Acute Lymphoblastic Leukemia in Adults. N. Engl. J. Med..

[47] Advani A.S., Moseley A.B., O’Dwyer K.M., Wood B.L., Park J.H., Wieduwilt M.J., Jeyakumar D., Yaghmour G., Atallah E.L., Gerds A.T. (2023). Long-Term Follow up for SWOG 1318: Combination of Dasatinib, Prednisone, and Blinatumomab for Older Patients with Philadelphia-Chromosome (Ph) Positive Acute Lymphoblastic Leukemia (ALL). Blood.

[48] Lang F., Pfeifer H., Brüggemann M., Hermann E., Serve H., Goekbuget N. (2024). A Multicentre, Randomized Trial in Adults with de novo Philadelphia Chromosome-Positive Acute Lymphoblastic Leukaemia to Assess the Efficacy of Ponatinib versus Imatinib in Combination with Low-Intensity Chemotherapy, to Compare End of Therapy with Indication for Stem Cell Transplantation versus Tyrosine Kinase Inhibitor, Blinatumomab, and Chemotherapy in Optimal Responders, and to Evaluate Blinatumomab in Suboptimal Responders (GMALL-EVOLVE). Oncol. Res. Treat..

[49] Zhang T., Zhu K., Zihong C., Lin R., Liu Q., Zhou H. (2023). Frontline Combination of 3 rd Generation TKI Olverembatinib and Blinatumomab for Ph+/Ph-like ALL Patients. Blood.

[50] Luskin M.R., Murakami M.A., Keating J.H., Wang E.S., McMasters M., Flamand Y., Winer E.S., Stahl M., Smith H., Jaeckle S.L. (2025). A phase I study of asciminib in combination with dasatinib, prednisone, and blinatumomab for Ph-positive acute leukemia in adults. J. Clin. Oncol..

[51] ClinicalTrials.gov. Testing the Use of Steroids and Tyrosine Kinase Inhibitors With Blinatumomab or Chemotherapy for Newly Diagnosed BCR-ABL-Positive Acute Lymphoblastic Leukemia in Adults. https://www.clinicaltrials.gov/study/NCT04530565.

[52] ClinicalTrials.gov. Sequential Treatment With Ponatinib and Blinatumomab vs Chemotherapy and Imatinib in Newly Diagnosed Adult Ph+ ALL. https://clinicaltrials.gov/study/NCT04722848.

[53] Lee D.W., Kochenderfer J.N., Stetler-Stevenson M., Cui Y.K., Delbrook C., Feldman S.A., Fry T.J., Orentas R., Sabatino M., Shah N.N. (2015). T cells expressing CD19 chimeric antigen receptors for acute lymphoblastic leukaemia in children and young adults: A phase 1 dose-escalation trial. Lancet.

[54] Maude S.L., Laetsch T.W., Buechner J., Rives S., Boyer M., Bittencourt H., Bader P., Verneris M.R., Stefanski H.E., Myers G.D. (2018). Tisagenlecleucel in Children and Young Adults with B-Cell Lymphoblastic Leukemia. N. Engl. J. Med..

[55] Shah B.D., Ghobadi A., Oluwole O.O., Logan A.C., Boissel N., Cassaday R.D., Leguay T., Bishop M.R., Topp M.S., Tzachanis D. (2021). KTE-X19 for relapsed or refractory adult B-cell acute lymphoblastic leukaemia: Phase 2 results of the single-arm, open-label, multicentre ZUMA-3 study. Lancet.

[56] Roddie C., Sandhu K.S., Tholouli E., Logan A.C., Shaughnessy P., Barba P., Ghobadi A., Guerreiro M., Yallop D., Abedi M. (2024). Obecabtagene Autoleucel in Adults with B-Cell Acute Lymphoblastic Leukemia. N. Engl. J. Med..

[57] Chiesa R., Georgiadis C., Syed F., Zhan H., Etuk A., Gkazi S.A., Preece R., Ottaviano G., Braybrook T., Chu J. (2023). Base-Edited CAR7 T Cells for Relapsed T-Cell Acute Lymphoblastic Leukemia. N. Engl. J. Med..

[58] Oh B.L.Z., Shimasaki N., Coustan-Smith E., Chan E., Poon L., Lee S.H.R., Yeap F., Tan L.K., Chai L.Y.A., Le Bert N. (2024). Fratricide-resistant CD7-CAR T cells in T-ALL. Nat. Med..

[59] Pan J., Tan Y., Shan L., Seery S., Deng B., Ling Z., Xu J., Duan J., Wang Z., Wang K. (2025). Allogeneic CD5-specific CAR-T therapy for relapsed/refractory T-ALL: A phase 1 trial. Nat. Med..

[60] Song Y., Chen S., Liu C., Chen L., Wang W., Wu B., Liang Y. (2023). Chemo-free maintenance therapy in adult T-cell acute lymphoblastic leukemia: A case report and literature review. Front. Pharmacol..

[61] Cassaday R.D., Garcia K.A., Fromm J.R., Percival M.M., Turtle C.J., Nghiem P.T., Stevenson P.A., Estey E.H. (2020). Phase 2 study of pembrolizumab for measurable residual disease in adults with acute lymphoblastic leukemia. Blood Adv..

[62] Luskin M.R., Shimony S., Keating J., Winer E.S., Garcia J.S., Stone R.M., Jabbour E., Flamand Y., Stevenson K., Ryan J. (2025). Venetoclax plus low-intensity chemotherapy for adults with acute lymphoblastic leukemia. Blood Adv..

[63] Schultz L.M., Jeyakumar N., Kramer A.M., Sahaf B., Srinagesh H., Shiraz P., Agarwal N., Hamilton M., Erickson C., Jacobs A. (2024). CD22 CAR T cells demonstrate high response rates and safety in pediatric and adult B-ALL: Phase 1b results. Leukemia.

[64] Liu S., Zhang X., Dai H., Cui Q., Cui W., Yin J., Li Z., Xue S., Yang C., Yang X. (2021). Tandem CD19/CD22 Dual Targets CAR-T Cells Therapy Obtains Superior CR Rate Than Single CD19 CAR-T Cells Infusion As Well As Sequential CD19 and CD22 CAR-T Cells Infusion for Relapsed/Refractory B-Cell Acute Lymphoblastic Leukemia Patients. Blood.

[65] Li L., Wang L., Liu Q., Wu Z., Zhang Y., Xia R. (2022). Efficacy and safety of CD22-specific and CD19/CD22-bispecific CAR-T cell therapy in patients with hematologic malignancies: A systematic review and meta-analysis. Front. Oncol..

[66] ClinicalTrials.gov. Daratumumab for MRD-Positive Acute Lymphoblastic Leukemia. https://www.clinicaltrials.gov/study/NCT03808610.

[67] Zhang J., Wang S., Zhang D., He X., Wang X., Han H., Qin Y. (2023). Nanoparticle-based drug delivery systems to enhance cancer immunotherapy in solid tumors. Front. Immunol..

[68] Tasian S.K., Loh M.L., Hunger S.P. (2017). Philadelphia chromosome-like acute lymphoblastic leukemia. Blood.

[69] Mikhael J., Fowler J., Shah N. (2022). Chimeric Antigen Receptor T-Cell Therapies: Barriers and Solutions to Access. JCO Oncol. Pract..

